# Supplementary material for: Inverse modeling, analysis and control of twin rotor aerodynamic systems with optimized artificial intelligent controllers
Source: PLoS One. 2025 May 19;20(5):e0322999. doi: 10.1371/journal.pone.0322999 (PMC12088051; doi:10.1371/journal.pone.0322999)
Supplement: Attachment — Submitted filename: PONE-D-24-45546_review1.pdf [file pone.0322999.s001.pdf]

This paper presents a novel approach to controlling Twin Rotor Aerodynamic Systems (TRAS) using an optimal inverse Radial Basis Function (RBF) neural network model, addressing the challenges posed by the high nonlinearity and coupling effects in TRAS, modeled as Multi-Input Multi-Output (MIMO) systems. The paper is well-written, but a few improvements are needed to enhance clarity and rigor. Below are my detailed comments:

Major Remarks:

1. **Real-Time Implementation and Robustness**: Could you provide more details on how the proposed approach (ASO with inverse RBF neural networks) improves traditional methods in practical scenarios? Specifically, how does it perform in real-time implementation and under system uncertainties? Empirical evaluation or case studies on these aspects would strengthen the paper's practical relevance.
2. **Figure Captions Consistency**: The captions in Figures 8, 10, 14, and 16 are not uniform. It is recommended to standardize the phrasing and style to ensure consistency and improve the overall readability and professionalism of the presentation.
3. **Clarification of Theorem 2 Proof**: The proof of Theorem 2 is well-structured, but the transition between equations (50) to (54) lacks clarity. Provide explicit reasoning for substitutions such as $ \hat{u}_p(t) $, $ P_{\phi} e(t) $, and $ D_{\phi} \dot{e}(t) $ would be helpful. Additionally, clarify the summation $ \sum |w_j| $ and its relationship to $ x_{\phi} $ in Equation (55), justify the inequality $ |A_{\text{new}}| \leq k |B_{\phi}| $ in Equation (56), and elaborate on how $ \dot{V} \leq 0 $ ensures stability via the Lyapunov criterion would strengthen the rigor of the proof.
4. **Comparison with Traditional Methods**: Could you elaborate on why the proposed approach outperforms traditional PID and hybrid methods ([11]-[14])? Specifically, how does the inverse RBF neural network, optimized using the ASO algorithm, better handle high system nonlinearities? A clear comparison of performance metrics like tracking accuracy, adaptability, and robustness would highlight the advantages of the proposed method.

Minor Remarks:

1. There is a typo in the constant after Equation 20. Please ensure that the constant ‘kbest’ is corrected for consistency throughout the paper.
2. Correct MATLAB notation in Section 10: “Simulation Results”.
3. Please correct the notation for the sample (n) to ensure consistency throughout the document wherever required.
