# Supplementary material for: Inverse modeling, analysis and control of twin rotor aerodynamic systems with optimized artificial intelligent controllers
Source: PLoS One. 2025 May 19;20(5):e0322999. doi: 10.1371/journal.pone.0322999 (PMC12088051; doi:10.1371/journal.pone.0322999)
Supplement: Attachment — Submitted filename: Response to Reviewers.pdf [file pone.0322999.s003.pdf]

Title: Inverse Modeling, Analysis and Control of Twin Rotor Aerodynamic Systems with Optimized Artificial Intelligent Controllers
Manuscript Number: PONE-D-24-45546

Dear Respected Reviewers,

First of all, we would like to express our sincere gratitude and appreciation to the valuable feedback provided by the Editor and Reviewers. Secondly, attached please find the revised manuscript "**Inverse Modeling, Analysis and Control of Twin Rotor Aerodynamic Systems with Optimized Artificial Intelligent Controllers**". Reviewers' comments were addressed in this version. We hope that it will meet the editors'/reviewers' expectation and be accepted for publication in **PLOS ONE Journal**.
The paper was originally submitted under **ID: PONE-D-24-45546** and the decision of the review process was as follow: "**MAJOR REVISION**".
We have carefully considered each comment provided by the reviewers and most the provided comments and suggestions were implemented in the revised manuscript. We believe that these comments and suggestions have improved the overall quality of the paper.
In the next few pages, we have provided a detailed response to each comment/suggestion provided by the reviewers. For clarity of exposition, all changes in the manuscript are highlighted in blue.

Yours sincerely,

Authors

First of all, we would like to thank the reviewers for their valuable comments and suggestions. Please find our detailed responses in blue.

********************* Reviews *********************

Reviewer 1:

1. Include experimental results on a TRAS since at the moment, the work is limited to the simulation environment only. At least, please include a discussion on, to what extent, are the simulation results can be mapped to control a real system?

Authors response: Thanks to the reviewer for his valuable feedback. The authors appreciate his suggestion to include experimental results on TRAS to provide a solid foundation for the work in this field. The results presented in this manuscript serve as a strong theoretical basis for the effectiveness of the proposed approach. Additionally, the simulation environment was designed to closely mimic real-world scenarios, including system uncertainties and disturbances, to ensure the robustness and adaptability of the proposed method. While real-time implementation is beyond the scope of this paper, we acknowledge its importance for practical validation. The ASO algorithm and RBF neural network are lightweight and can be implemented on embedded systems. Moreover, the proposed method can be integrated with standard control hardware, such as microcontrollers or FPGAs, for real-time deployment. The authors look forward to incorporating these enhancements into their future work as illustrated in Section 12 of the revised manuscript (please refer to page 25).

2. More rigorous analysis of error is required e.g. include results on other parameters such as ITAE, ISE, etc.

Authors response: Thanks to the Reviewer for his insightful suggestion to include a more rigorous analysis of error using performance indices such as ITAE, ISE and ITSE. We agree that this will strengthen the robustness of our results. In response to the reviewer's suggestion, we have calculated the ITAE, ISE and ITSE for each controller used in the simulation. These metrics were evaluated and are now included in the revised manuscript, as showed in Table 5 (please refer to page 19 of the revised manuscript).

3. It is mentioned that a TRAS can find potential to be used as a test bed for analysing the performance of control algorithms for helicopters, UAVs, etc. Include related studies exploiting this feature of TRAS such as; 'Differentiator- and Observer-Based Feedback Linearized Advanced Nonlinear Control Strategies for an Unmanned Aerial Vehicle System' and 'Adaptive optimal control of twin-rotor helicopter system; Meta-heuristics and Lyapunov based approach'.

Authors response: Thanks to the Reviewer for his valuable suggestion to include further related studies that highlight the use of the TRAS as a test bed for analyzing control algorithms for helicopters, UAVs, and similar systems. We agree that this will support our work. Thus, following the reviewer's recommendation, this issue is considered in the revised manuscript. Specifically, we have added the following studies to the revised manuscript:

- 'Differentiator- and Observer-Based Feedback Linearized Advanced Nonlinear Control Strategies for an Unmanned Aerial Vehicle System'
- 'Adaptive optimal control of twin-rotor helicopter system; Meta-heuristics and Lyapunov based approach'

These studies have been cited in the Introduction Section and added to the Reference Section (please refer to pages 2 and 25 of the revised manuscript).

4. Include the Paper Outlines at the end of Section 1 (Introduction).

Authors response: Thanks to the Reviewer for noting this. The Reviewer's comment alerted us that the Paper Outlines are missing. This issue is addressed in the revised manuscript by adding the Paper outlines at the end of the Introduction section (please refer to page 3 of the revised manuscript).

5. Both words are used in the paper; TRAS and TRMS. I suppose they are same? If yes, please make them consistent.

Authors response:

Thanks to the Reviewer for his comment. The Reviewer is correct in noting that both two term TRAS and TRMS are used within the manuscript, which are the same. Therefore, the authors modified the manuscript to only used the acronym TRAS (please refer to page 16 of the revised manuscript).

6. PD controller is used. What is the rationale for that? because the Integral component could have helped to further improve the Steady State error.

Authors response: Thanks to the reviewer for his valuable comment. In response, to explain the rationale for using a PD controller instead of a PID controller, we need to demonstrate why the integral component was excluded despite its capability to improve steady-state error, and how the PD controller's design achieves our goal. The decision was based on the following considerations:

1- Our design focuses on fast response, stability and minimizing steady state error.
2- As we know, the Proportional part ensures a fast response, while the Derivative component improves stability by damping oscillations and reducing overshoot. Therefore, the PD controller makes the system faster with minimal overshoot.
3- While the Integral part is traditionally used to eliminate steady-state error, it can lead to integral windup in systems with high nonlinearities or saturation limits, causing instability or excessive overshoot.
4- In Section 10, we prove the stability of the proposed controller using only a PD controller, which simplifies our system compared to a PID controller.
5- The RBF network dynamically adjusts its parameters to compensate for system nonlinearities and disturbances which effectively reduces steady-state error.
6- The ASO algorithm ensures that the controller's parameters are optimally tuned, further minimizing steady-state error.

In conclusion, in our proposed approach, the inverse RBF neural network, optimized by ASO, ensures that steady-state error is minimized without the need for an Integral part.

7. Update your literature review on control of TRAS including notable references such as 'A flexible mixed-optimization with H∞ control for coupled twin rotor MIMO system based on the method of inequality (MOI) - An Experimental Study'.

Authors response: Thanks to the Reviewer for his valuable suggestion to update the literature review on the control of TRAS and we appreciate his recommendation to include the notable reference:

'A flexible mixed-optimization with H∞ control for coupled twin rotor MIMO system based on the method of inequality (MOI) - An Experimental Study'.

We agree that this reference is highly relevant to our work and will strengthen the context of our literature review. It has been cited in the Introduction Section of the revised manuscript, and its findings have been highlighted. It is added to the Reference Section (please refer to pages 2 and 26 of the revised manuscript).

8. Include the stability analysis of the proposed hybrid control law.

Authors response: Thanks to the Reviewer for his comment. Section 10 of the revised manuscript provides the closed-loop stability analysis of the proposed hybrid control law using the Lyapunov stability theorem (please refer to pages 16-17 of the revised manuscript).

9. The simplicity feature of a PID-based control law, mentioned in Section 1 (Introduction) could benefit from the reference 'Embedded control system for autarep - a novel autonomous articulated robotic educational platform'.

Authors response: Thanks to the Reviewer for the valuable suggestion to include the notable reference:

"Embedded control system for autarep - a novel autonomous articulated robotic educational platform".

We agree that incorporating this reference will further support and strengthen the discussion on the simplicity of the PID-based control law. In response to the reviewer's recommendation, we have revised the Introduction and Reference Sections to include this reference (please refer to pages 2 and 26 of the revised manuscript).

10. At the end of the Abstract, please include 1-2 sentences to quantitatively summarise the key findings of your proposed hybrid control algorithm.

Authors response: Thanks to the reviewer for his valuable comment. Following the Reviewer's advice, we have enhanced the abstract by adding, at the end, a quantitative summary of the key findings of our proposed hybrid control algorithm (please refer to page 1 of the revised manuscript).

11. It is mentioned that FOPID is better and smoother than the traditional PID controllers. This statement could be crisper if supported with a literature work such as 'Robust MPPT control of stand-alone photovoltaic systems via adaptive fractional-order PID controller with self-adjusting fractional orders'.

Authors response: We appreciate the reviewer's insightful suggestion to strengthen the statement regarding the superiority of FOPID over traditional PID controllers. In response to this suggestion, we have revised the manuscript and included the suggested reference (please refer to pages 2 and 26 of the revised manuscript).

'Robust MPPT control of stand-alone photovoltaic systems via adaptive fractional-order PID controller with self-adjusting fractional orders'

Which provides evidence from the literature to support our assertion that FOPID offers better and smoother performance compared to traditional PID controllers.

12. Does the novelty of the proposed work lie in proposing a hybrid controller (combination of PD with inverse RBF)? Please explicitly mention the novelty element.

Authors response: Thanks to the Reviewer for his important comment regarding the novelty of our proposed work. The Reviewer is correct in noting that the novelty of our work lies in proposing a hybrid controller that combines a Proportional-Derivative (PD) controller with an inverse Radial Basis Function (RBF) neural network model-based ASO identification for TRAS. This hybrid approach exploits the stability and simplicity of the PD controller while utilizing the adaptive and learning capabilities of the inverse RBF to handle system nonlinearities and uncertainties. To the best of our knowledge, this specific combination has not been extensively explored in the context of TRAS (please refer to pages 2-3 of the revised manuscript, where a clear statement is provided).

13. In Table 1, include a new column 'Description' where each parameter is briefly mentioned what it is? Also, the units may be moved to a new column.

Authors response: We appreciate the Reviewer's suggestion to enhance the clarity and organization of Table 1. In response, we have added a 'Description' column and separated the units into a new column, as illustrated in Table 1 on page 5 of the revised manuscript. The updated table now provides a clearer and more detailed presentation of the parameters, their descriptions, and their respective units.

14. In Figure 4, include the signs to each feedback /feed-forward loop (the is the inputs to each summer should be assigned a sign). I suppose it is positive?

Authors response: Thanks to the Reviewer for noting this. The reviewer's comment alerted us to that the signs of the feedback and feed-forward loops were missing. In response, we have addressed this issue by revising Figure 4 to include the signs for each input to the summing junctions (please refer to Figure 4 on page 12 of the revised manuscript).

15. In Table 2, write 0 with .0561

Authors response: Thanks to the Reviewer for his attention to detail. The authors have made the required change (please refer to Table 2 on page 15 of the revised manuscript).

16. Please improve the quality of all Figures.

Authors response: Thanks to the Reviewer for the valuable suggestion to improve the quality of the figures in our manuscript. Following the Reviewer's recommendation, all Figures have been reproduced in the revised manuscript to ensure better quality.

17. Also, thoroughly proofread the paper for typos and other linguistic improvements.

Authors response: To address the Reviewer's concern, all the textual corrections have been made, and the manuscript was proofread by a native English speaker from Canada. Also, the manuscript has been subjected to thorough revisions, and the typographical and grammatic issues have been essentially eliminated.

Reviewer 2:

This paper presents a novel approach to controlling Twin Rotor Aerodynamic Systems (TRAS) using an optimal inverse Radial Basis Function (RBF) neural network model, addressing the challenges posed by the high nonlinearity and coupling effects in TRAS, modeled as Multi-Input Multi-Output (MIMO) systems. The paper is well-written, but a few improvements are needed to enhance clarity and rigor. Below are my detailed comments:

Major Remarks:

1. **Real-Time Implementation and Robustness:** Could you provide more details on how the proposed approach (ASO with inverse RBF neural networks) improves traditional methods in practical scenarios? Specifically, how does it perform in real-time implementation and under system uncertainties? Empirical evaluation or case studies on these aspects would strengthen the paper's practical relevance.

Authors response: We appreciate the reviewer's input. In response, we would like to explain that a similar comment was raised by the first Reviewer, and the answer to this comment is provided in our first response.

2. **Figure Captions Consistency:** The captions in Figures 8, 10, 14, and 16 are not uniform. It is recommended to standardize the phrasing and style to ensure consistency and improve the overall readability and professionalism of the presentation.

Authors response: Thanks to the reviewer for pointing out the inconsistency in the captions of Figures 8, 10, 14, and 16. We have revised the captions to ensure uniformity in phrasing and style, improving readability and professionalism (please refer to pages 20, 21, 23-24 of the revised manuscript).

3. **Clarification of Theorem 2 Proof:** The proof of Theorem 2 is well-structured, but the transition between equations (50) to (54) lacks clarity. Provide explicit reasoning for substitutions such as $ \hat{u}_p(t) $, $ P_{\phi} e(t) $, and $ D_{\phi} \dot{e}(t) $ would be helpful. Additionally, clarify the summation $ \sum

|w_j| $ and its relationship to $ x_{\phi} $ in Equation (55), justify the inequality $ |A_{\text{new}}| \leq k |B_{\phi}| $ in Equation (56), and elaborate on how $ \dot{V} \leq 0 $ ensures stability via the Lyapunov criterion would strengthen the rigor of the proof.

Authors response: Thanks to the reviewer for his valuable comment.

1- Following the reviewer's recommendation, we have enhanced the technical understanding of Equations (50) to (54) by providing more details on its mathematical formulation. The equations are clearly presented and defined. Additionally, the relationship between the equations is provided. The details mathematical transition between equations (50) to (54) is illustrated by Equations (50) to (59).
2- Moreover, the inequality has been justified.
3- The relationship between $w_{\emptyset j}$ and $x_{\emptyset}$ comes from the fact that:

$$V_1 = \frac{1}{2} x_{\emptyset}^T x_{\emptyset}$$

$$\dot{V}_1 = x_{\emptyset}^T \dot{x}_{\emptyset}$$

Substituting Equation (10) gives:

$$\dot{V}_1 = x_{\emptyset}^T [A_{\emptyset} x_{\emptyset} + B_{\emptyset} u_{\emptyset}]$$

where:

$$u_{\emptyset} = \left(\hat{u}_{p\emptyset}(t) + u_{PD}(t)\right)$$

and $\hat{u}_{p\emptyset} = w_{\emptyset_0} + \sum_{j=1}^{H} w_{\emptyset j} \emptyset_j \left(\left\| x_{\emptyset} - \mu_{\emptyset_j} \right\| \right)$

4. **Comparison with Traditional Methods:** Could you elaborate on why the proposed approach outperforms traditional PID and hybrid methods ([11]-[14])? Specifically, how does the inverse RBF neural network, optimized using the ASO algorithm, better handle high system nonlinearities? A clear comparison of performance metrics like tracking accuracy, adaptability, and robustness would highlight the advantages of the proposed method.

Authors response: Thanks to the Reviewer for his important comments, which alerted us that more discussion is required regarding:

1- The ability of the inverse RBF neural network optimized by ASO to handle high system nonlinearities.

The inverse RBF neural network represents the main component of the proposed controller. Unlike traditional PID controllers, which rely on fixed gains and linear control laws, the inverse RBF neural network can adapt to system nonlinearities. Specifically, it can approximate complex nonlinear functions, allowing it to model and compensate for high system nonlinearities, like TRAS, that traditional PID controllers cannot effectively handle. The network has the ability to learn and adjust its parameters to ensure optimal performance, even in the presence of uncertainties or changes in operating conditions. Accordingly, the ASO algorithm,

as an optimization algorithm, is used to optimize the parameters of the inverse RBF neural network. As a result, this algorithm enhances the controller's performance because the ASO algorithm acts as a global optimizer to find the optimal parameters. Moreover, it dynamically adjusts the balance between exploration (searching for new solutions) and exploitation (refining existing solutions) to ensure robust and efficient optimization.

Thus, following the reviewer's recommendation, this issue is further elaborated in the revised manuscript (please refer to pages 1-2 of the revised manuscript).

2- Provide a clear comparison of performance metrics to highlight the advantages of our proposed method.

Table 5 presents the transient specifications and integral performance indices of TRAS for each controller. The proposed controller exhibits an 88.3% faster rise time, a 96.0% faster settling time, and a 93.8% lower overshoot for the Yaw model, along with a 42.8% faster rise time, a 73.9% faster settling time, and an 86.8% lower overshoot for the Pitch model compared to the FOPID controller. In comparison to the PSO-PID controller, the proposed controller shows a 36.2% faster rise time, an 86.7% faster settling time, and a 59.7% lower overshoot for the Yaw model, as well as a 58.4% slower rise time, a 59.9% faster settling time, and a 71.2% lower overshoot for the Pitch model. These results show that the proposed controller, due to the adaptive nature of the RBF network, demonstrates faster adaptation, as evidenced by its lower rise and settling times. Furthermore, there are significant reductions in the integral performance indices (e.g., IAE, ITAE, ISE and ITSE) for the proposed controller compared to the FOPID and PSO-PID controllers. This indicates that the proposed controller achieves lower tracking errors by dynamically compensating for system nonlinearities. For example, compared to FOPID and PSO-PID, the proposed algorithm reduces the IAE performance index by 93.0% and 41.3% for the Yaw model, and by 97.7% and 94.7% for the Pitch model, respectively. Overall, the proposed controller demonstrates noticeable improvements over both FOPID and PSO-PID.

Thus, following the reviewer's recommendation, this issue is further elaborated and discussed (please refer to pages 19 and 24 of the revised manuscript).

Minor Remarks:

1. There is a typo in the constant after Equation 20. Please ensure that the constant ‘kbest’ is corrected for consistency throughout the paper.

Authors response: Thanks to the Reviewer for his attention to detail. In response to the reviewer's comment, we have corrected the typo in the constant '*kbest*'. Also, the authors have carefully reviewed the entire manuscript to ensure that '*kbest*' is used consistently in all relevant sections.

2. Correct MATLAB notation in Section 10: “Simulation Results”.

Authors response: Thanks to the Reviewer for his comment. The authors have made the required change (please refer to page 18 of the revised manuscript).

3. Please correct the notation for the sample (n) to ensure consistency throughout the document wherever required.

Authors response: Thanks to the Reviewer for his valuable comment. Following the Reviewer's recommendation, the authors have corrected the notation for the simple '*n*' throughout the entire manuscript to ensure consistency.
